# Physiotherapy-led telehealth and exercise intervention to improve mobility in older people receiving aged care services (TOP UP): protocol for a randomised controlled type 1 hybrid effectiveness-implementation trial

**DOI:** 10.1136/bmjnph-2022-000606

**Published:** 2023-11-14

**Authors:** Rik Dawson, Marina Pinheiro, Vasikaran Nagathan, Morag Taylor, Kim Delbaere, Juliana Olivera, Abby Haynes, Jenny Rayner, Leanne Hassett, Catherine Sherrington

**Affiliations:** 1 Sydney Musculoskeletal Health, Faculty of Health and Medicine, School of Public Health, The University of Sydney Institute for Musculoskeletal Health, Sydney, New South Wales, Australia; 2 Sydney Local Health District, Sydney, New South Wales, Australia; 3 Faculty of Health and Medicine, School of Public Health, The University of Sydney, Sydney, New South Wales, Australia; 4 Department of Geriatric Medicine, Sydney Local Health District, Sydney, New South Wales, Australia; 5 Falls, Balance and Injury Research Centre, Neuroscience Research Australia, Randwick, New South Wales, Australia; 6 Faculty of Medicine, University of New South Wales, Sydney, New South Wales, Australia

**Keywords:** Randomised Controlled Trial, Telehealth, Physiotherapy, Aged Care

## Abstract

**Introduction:**

Deteriorating mobility and falls reduce independence and quality of life for older people receiving aged care services. This trial aims to establish effectiveness on the mobility of older people, and explore cost-effectiveness and implementation of a telehealth physiotherapy programme.

**Method and analysis:**

This type 1 hybrid effectiveness-implementation randomised controlled trial will involve 240 people aged 65+ years receiving aged care services in community or residential settings. Participants will be randomised to either: (1) the Telehealth Physiotherapy for Older People (TOP UP) Program or (2) a wait-list control group. The 6-month intervention includes 10 physiotherapy sessions delivered by videocall (Zoom). The intervention will include the local support of an aged care worker and online exercise resources. Primary outcome is mobility at 6 months post randomisation measured by the Short Physical Performance Battery. Secondary outcomes include rate of falls, sit-to-stand, quality of life, and goal attainment at 6 months after randomisation. Regression models will assess the effect of group allocation on mobility and the other continuously scored secondary outcomes, adjusting for baseline scores. The number of falls per person over 6 months will be analysed using negative binomial regression models to estimate between-group differences. An economic analysis will explore the cost-effectiveness of the TOP UP programme compared with usual care. Implementation outcomes and determinants relating to the intervention’s reach, fidelity, exercise dose delivered, adoption, feasibility, acceptability, barriers and facilitators will be explored using mixed methods.

**Conclusion:**

This is the first trial to investigate the effectiveness, cost-effectiveness and implementation of a physiotherapy intervention in aged care delivered solely by telehealth internationally. The study has strong aged care co-design and governance and is guided by steering and advisory committees that include staff from aged care service providers and end-users. Trial results will be disseminated via peer-reviewed articles, conference presentations and lay summaries.

**Trial registration number:**

The trial is registered with the Australian New Zealand Clinical Trials Registry (ACTRN 12621000734864).

**What is already known on this topic**

- Telehealth physiotherapy is an effective health service in primary care.
- Exercise programmes improve mobility for older people.

**What this study adds**

- Establish the effectiveness of telehealth physiotherapy in aged care on mobility.
- Explore the impact of online resources on exercise dose.

**How this study might affect research, practice or policy**

- Our implementation analysis will guide the adoption of telehealth physiotherapy in aged care.

## Introduction

Older people receiving aged care services have a high prevalence of multimorbidities, dependency in activities of daily living and mobility limitations.^1^ Mobility disability (ie, difficulty or inability to walk and transfer) is a common and a serious form of physical disability.^2^ Mobility disabilities affect approximately 35% of people aged 70 years and older, and the majority of people aged over 85 years.^3^ Mobility disability is predictive of adverse health outcomes, including death.^4^

Exercise is recommended for those who have mobility disability.^5^ The 2020 update of the World Health Organisation’s (WHO) physical activity guidelines call for older people to undertake 150–300 min/week of moderate-intensity activity, muscle strengthening and exercises targeting balance.^6^ This 2020 update marked the release of specific guidelines for people living with disability.^7^ These guidelines specify that people living with disability should, where possible, undertake the same amount and types of physical activity (including balance, functional strength and endurance activities) as is recommended for the general population.

Physiotherapists are well placed to deliver suitable exercise programmes for older people with mobility disability. Exercise-based interventions designed and delivered face to face by physiotherapists in aged care settings have been found to improve mobility.^8^ However, in Australia, there is a lack of suitably-trained exercise professionals to deliver these interventions, particularly in regional areas.^9^

Telehealth could provide a cost-effective way to increase older people’s access to physiotherapists and has been successfully used in other populations and contexts.^10^ The WHO defines telehealth as the delivery of healthcare services where patients and providers are separated by distance and where health professionals use information communication technologies for the exchange of information for the diagnosis and treatment of diseases and injuries, education, research and evaluation.^11^ The COVID-19 pandemic has rapidly accelerated familiarity with, and infrastructure required for, telehealth provision in aged care.^12^ However, no randomised controlled trials to date have been designed to examine the effect or implementation of telehealth physiotherapy on mobility and falls for older people receiving aged care services.

To address this evidence gap, we will conduct a type 1 hybrid effectiveness-implementation randomised controlled trial to evaluate the effectiveness, cost-effectiveness and implementation of the Telehealth Physiotherapy for Older People (TOP UP) Program. The TOP UP Study tests a 6-month telehealth physiotherapy-led exercise programme, which aims to improve mobility compared with a wait-list control of usual care on older people aged 65+ years receiving aged care services in their home or in residential aged care. Additionally, we aim to measure the effects of telehealth physiotherapy on secondary outcomes of fall rate, sit-to-stand ability, health-related quality of life and goal attainment at 6 months. We will also evaluate the cost-effectiveness of the telehealth physiotherapy programme and if proven effective, explore implementation to guide future scale-up.

## Methods and analysis

### Study design and setting

**Figure 1:**
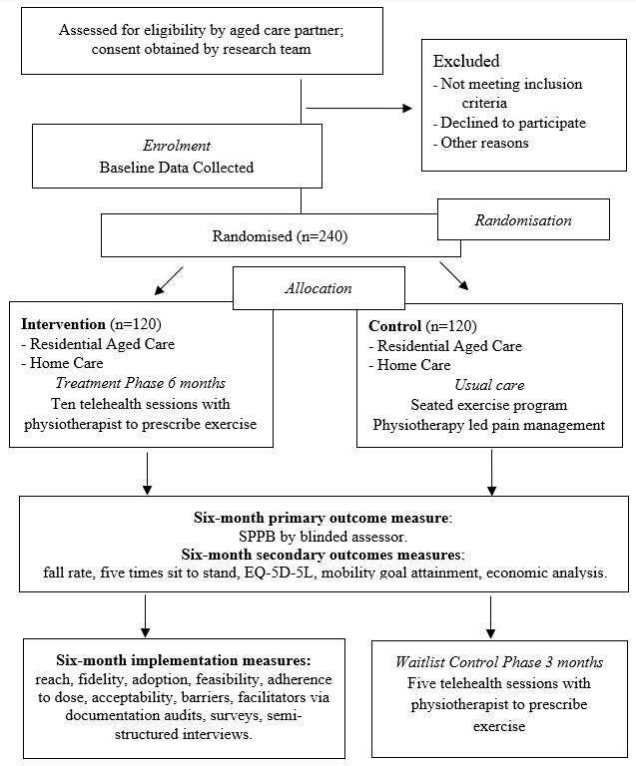
Trial design.

We will conduct a two-arm parallel, pragmatic, type 1 hybrid effectiveness-implementation randomised controlled trial. Trial design is illustrated in figure 1. Trial and protocol reporting will be guided by the CONsolidated Standards Of Reporting Trials statement,^13^ the Standard Protocol Items: Recommendations for Interventional Trials statement,^14^ the Template for Intervention Description and Replication checklist)^15^ and the Consensus on Exercise Reporting (CERT) guidelines.^16^

### Participants

#### Inclusion criteria

People eligible for inclusion are aged 65 years and over; living in the community or residential aged care receiving Commonwealth-funded aged care services; willing to use a mobile tablet device to video-conference with a physiotherapist; willing to exercise for 2 hours/week; and have sufficient sensory, neurological, cognitive and English language skills for exercise and video-based interventions as determined by their aged care service provider.

#### Exclusion criteria

Potential participants will be excluded if they have severe cognitive impairment as measured by a score of ten or less on the Modified Telephone Interview for Cognitive Status^17^; are unable to walk 10 m with or without a walking aid; are currently participating in a balance and strengthening exercise programme designed by a physiotherapist; and have life expectancy less than 6 months as determined by their aged care service providers.

### Recruitment and consent

The aged care services will generate a list of potential participants using a standardised screening form. Potential eligible participants will receive information about the study through written participant information sheets and will have the opportunity to talk to their aged service provider and/or research team. Eligible participants will be enrolled once they have signed the consent form. If a potential participant is considered by their aged care provider not to have full capacity to provide consent, the potential participant’s person responsible will be asked to provide consent. Participants’ General Practitioner will be sent a letter explaining their participation in the trial allowing the doctor to discuss any concerns with the research team and suggest withdrawal of participants from the study.

### Randomisation

Participant baseline data will be collected before randomisation. Two hundred and forty participants will be randomly assigned (1:1) to either the telehealth physiotherapy TOP UP exercise programme or to the wait-list control group. The trial will use a centralised web-based randomisation system using Research Electronic Data Capture (REDCap) within The Sydney Local Health District license to ensure concealment of group allocation. A research investigator not involved in baseline assessment measures or recruitment of facilities will develop a randomisation schedule. They will use a computer-generated random number schedule with randomly permuted block sizes of two and four. Allocation to either the intervention group or the control group will be stratified according to the participants’ place of residence (at home receiving aged care services or living in residential aged care).

### Intervention

The TOP UP Study will use telehealth to prescribe an individualised exercise programme, deliver health coaching and technology training by a physiotherapist supported and supervised by a trained care worker called TOP UP Coaches (TCs). A computer or tablet will be used in the physiotherapist sessions using the Zoom videoconference application, for access to the TOP UP a website where participants can follow several progressive strengthening and balance exercise videos produced by the research team, and access to the *StandingTall* exercise app that delivers progressive balance exercise.^18^

Participants allocated to the TOP UP exercise programme will be invited to participate in ten 30-to-60-minute physiotherapy assessments using Zoom over 6 months. The initial assessment occurs in week 1 with the subsequent assessments scheduled to occur weeks 3, 5, 7, 9, 12, 16, 20, 22 and 24. The sessions will be conducted by Australian registered physiotherapists who have a minimum of 3 years aged care experience. The physiotherapists will create an individualised, progressive exercise programme based on the Otago Exercise Program (OEP).^19^ It will be delivered in the participant’s home or their residential aged care facility. The OEP is a series of progressive balance and lower limb resistive strengthening exercises that allows the treating physiotherapist to prescribe the level of intensity and pace of the programme. See table 1 for details of the exercise programme reported according to the CERT guidelines.^16^

**Table 1:** TOP UP strengthening and balance exercises and programme levels

Seated warm up and cool down: seated marching 1 min, neck and trunk gentle rotations, upper limb gentle range of movement exercise including ×10 repetitions (reps) shoulder elevation, elbow and wrist flexion and extension, ankle dorsiflexion and plantarflexion ×10 reps				
Strengthening exercises				
Seated knee extension	All 4 levels Ankle cuff weights used to provide resistance to the muscles and 10 reps of each exercise per set: building up to 3 sets of 10, hold support as required			
Standing knee flexion				
Standing hip abduction				
Standing ankle plantarflexion	Level 1: 10 reps, hold support Level 2: 2×10 reps, hold support		Level 3, 2×10 reps, hold support	Level 4, 2×10 reps, no support
Standing ankle dorsiflexion	Level 3 10 repetitions, hold support, 2 sets		Level 4 2×10 reps, no support, 2 sets	
Standing balance exercises				
	Level 1	Level 2	Level 3	Level 4
Backwards walking		10 steps, 2 reps, hold support	10 steps, 4 reps, minimal support	10 Steps, 4 reps, no support
Walking and turning around		Walk in a figure 8 twice, use walking aid if required	Walk in a figure 8 twice, minimal support	Walk in a figure 8 twice, no support
Sideways walking		10 steps, 2 reps, hold support	10 steps, 4 reps, minimal support	Walk 10 steps, no supports, 4 sets
Tandem stance	10 s, hold support	10 s, hold support	10 s, minimal support	10 s, no support
Heel-toe walk			Walk 10 steps, minimal support, 2 sets	Walk 10 steps, no support, 2 sets
One-leg stand		10 s, hold support	10 s, minimal support	30 s, no support
Heel walking			Walk 10 steps, hold supports, 4 sets	Walk 10 steps, no support, 4 sets
Toe walking			Walk 10 steps, hold support, 4 sets	Walk 10 steps, no support, 4 sets
Heel-toe walking backward			Walk 10 steps, hold support, 2 sets	Walk 10 steps, no support, 2 sets
Sit to stand	5 reps, 1 to 2 sets, 2 hands for support	10 reps, 2 hands for support	10 reps, 1 hand leading to no hands, 2 sets	10 reps, no support, 2 sets

Participants will be advised to complete up to 2 hours of exercise per week for the study based on previous research shown to improve mobility and reduce falls.^8^ Exercise dosage will be prescribed to accommodate comorbidities and cognitive impairments to minimise the risk of harm and will be tailored at the initial and each subsequent assessment. Participants will be asked to complete 2–3 sets of 10 repetitions for each exercise at a self-determined ‘moderate’ intensity, defined as 12 to 14 out of 20 using the Borg Scale of Perceived Exertion and the intensity will be progressed by the physiotherapist accordingly.^20^ The balance exercises will also be progressed by reducing hand support, reducing the base of support in standing, increasing the number of repetitions, and/or increasing the time in certain balance positions. Participants and TCs will record adherence to their exercise programme in an exercise diary that forms part of the participant booklet. More detail is provided in table 2.

**Table 2:** Intervention description using the Template for Intervention Description and Replication (TIDieR) checklist

Brief name	Telehealth physiotherapy-led exercise (TOP UP) Study to improve mobility in older people receiving aged care services.
2.Why	Older people receiving aged care services have a high prevalence of mobility disability and a high rate of falls. Physiotherapy-led exercise programmes that increase leg strength and challenge balance are proven to improve mobility and reduce falls. Telehealth is emerging as an effective method to deliver physiotherapy to improve access in regional areas and during COVID-19.
3.What materials	Participants allocated to the TOP UP exercise programme will be provided with a mobile tablet with internet connectivity to access Zoom, online exercise videos and the StandingTall app. Participants will receive a booklet comprising descriptions of their home exercise programme based on the Otago exercise programme, an exercise and falls calendar, ankle weights (1 and 2 kg), details on how to access and connect to the online exercise programmes, and balance support such as a sturdy dining chair, kitchen bench, parallel bars or wall bar.
4.What procedures	TOP UP physiotherapists will deliver balance and strength exercise prescription advice and health coaching using telehealth. Participants will be supported to access zoom to videoconference with a physiotherapist and aim to exercise for 2 hours/week supported by TOP UP Coaches (trained care staff of the participant’s aged care service provider). The research team will provide the physiotherapists and TOP UP Coaches with a 2-hour training session on the study protocol at the beginning of the study.
5.Who provides	The intervention will be conducted by either the research team’s physiotherapists or other registered physiotherapists employed by the study’s aged care partners. All physiotherapists will have 3 years+ experience in working in aged care supervising care staff. TOP UP Coaches will be selected by the aged care service providers from their interested pool of care workers.
6.How	TOP UP Coaches will support the participant to gain access to the technology for the Zoom physiotherapy sessions, lead group exercise classes in residential aged care, and supervise weekly the individual exercise programmes using the participant exercise booklet, online exercise videos, and StandingTall app. Participants allocated to the wait-list control group will receive a similar 3-month intervention once the trial is completed.
7.Where	The TOP UP Study will be delivered in the participant’s home or in the residential aged care facility where they live. Participants will be recruited from aged care service providers that deliver residential and home care services across metropolitan and regional areas in Australia.
8.When and how much	The 6-month intervention will include 10 Zoom sessions where the physiotherapists will devise moderate-intensity exercise programme and use health coaching principles to encourage participants to exercise for 2 hours/week. The participants can follow their exercise programme via exercise sheets in the participant booklet, follow 20–30 min exercise videos on the TOP UP website, attend group exercise programmes, or follow the StandingTall app. The programme will focus on standing balance and strength exercises. Participants will be provided with 30 min of supervised exercise with their TOP UP Coach each week to support the programme dose and safety.
9.Tailoring	The exercise programme will be tailored to the individual’s capabilities and comorbidities by a physiotherapist at assessment and at subsequent assessments. The physiotherapist will introduce supervised and unsupervised exercise and introduce online exercise resources that challenge balance and lower limb strength when appropriate.

Participants’ exercise dose is supported using different exercise resources prescribed by the physiotherapist. These include following their exercise programme with an exercise booklet, following online pre-recorded 15–30-min exercise videos on the TOP UP website, participate in individual or group exercise programmes led by TCs or follow their exercise programme on the *StandingTall* app. Access to online exercise programmes and unsupervised exercise will be introduced after the treating physiotherapist has completed a risk assessment.

### Behavioural change support

A key component of this intervention will be the behavioural change support delivered by the treating physiotherapist and TCs to each intervention participant over the trial period.^21^ The research team will conduct a 2-hour preintervention protocol training to treating physiotherapists and TCs informed by Michie’s COM-B model (table 3) and relevant Behaviour Change Taxonomy techniques^22 23^ to enhance participant’s motivation to adhere to their prescribed exercise programme.

**Table 3:** Trial intervention and the Capability,Opportunity and Motivation - Behavioural system (COM-B) for enhancing behaviour change^22 23^

Component	Definition	Behaviour change techniques
Capability	Individual’s psychological and physical capacity for engaging with exercise and telehealth including knowledge and skills.	Goal setting: development of SMART goals (specific, measurable, achievable, relevant, and timely). Action planning: encourage participants what, when, how, where they will exercise to assist with habit formation. Self-monitoring: encourage participants to keep track of their exercise programmes through use of diaries. Graded exercise: ensure that programme is achievable and progressive.
Opportunity	Factors outside the individual that enable or prompt behaviour.	Behavioural rehearsal/prompt cues/repetition: provision of exercise videos to follow with repetitive and detailed physiotherapy exercise advice. Instructions on how to perform a behaviour: provision of telehealth equipment and training to access physiotherapy advice. Social support and reward: TCs to assist with safe exercise delivery and socialisation opportunities.
Motivation	Cognitive processes that energise and direct behaviour, that is, goals, decision-making, habits, emotional responses.	Feedback on exercise performance and behaviours/ celebrate programme success: physiotherapists to provide feedback on participants’ performance, help them understand their progress and assist them to make necessary adjustments to their routine. Modelling: use of older people in printed and online exercise resources. Motivational interviewing/verbal persuasion: physiotherapist and TCs engage participants in a collaborative and empathetic conversation to strengthen their motivation and commitment to the exercise programme.

### Wait-list control group

Participants allocated to the wait-list control group will continue with usual care regarding their exercise and physiotherapy programmes in the first 6-month phase of the trial. This will include any pain management programmes delivered as part of the Aged Care Funding Instrument, any seated group exercise classes or mobility programmes delivered by the aged care service. These participants will be offered a similar 3-month intervention after the follow-up assessment.

### Data collection

Demographic data, questionnaires and physical assessments will be recorded by research physiotherapists blinded to group allocation at baseline and 6 months after randomisation. All outcomes will be assessed by research physiotherapists who will be trained in the conduct of the outcome assessment and are unaware of group allocation. Where face-to-face data collection is not possible, Zoom will be used with non-blinded TCs acting as local support under the supervision of the research physiotherapist to ensure safe and accurate data collection. Prior to the follow-up assessments, participants and TCs will be instructed not to inform the assessor of the their group assignment.

All intervention delivery costs including staff and equipment costs and all health service utilisation will be collected during the study period. The health service utilisation, falls and exercise data will be recorded by home care participants in their self-reported exercise diary and falls and health utilisation calendars. This data will be posted back to the research team by our aged care partners at the completion of the trial. In residential aged care this data will be recorded by TCs in the online documentation system. All data will be collected and uploaded into the REDCap database by a blinded member of our research team via auditing the participant’s online medical records and calendars.

All qualitative data will be collected by experienced research assistants using questionnaires and semistructured interviews via zoom. The implementation data collection related to the intervention’s reach, fidelity, dose delivered, adoption, feasibility and exercise adherence measures will be conducted by research assistants via audits of participants’ exercise diaries and physiotherapy notes.

### Outcomes

All outcomes will be measured in the intervention and control groups at baseline and 6 months after randomisation.

#### Primary outcome

Mobility will be measured by the 12-point *Short Physical Performance Battery (SPPB*) test score. The SPPB measures standing balance, gait (2.44 m timed walk), and timed sit-to-stand (five repetitions). The SPPB has predictive validity showing a gradient of risk for mortality, nursing home admission and disability.^24^

#### Secondary outcomes

The secondary outcomes will include: (1) rate of falls using the internationally recognised fall definition: ‘an unexpected event in which the participant comes to rest on the ground, floor, or lower level, as a result of a loss of balance”^25^; (2) five times sit-to-stand test will be analysed as an standalone predictor of future disability in older people^26^; (3) quality of life will be measured by the EuroQol 5 dimensions five level health questionnaire (EQ-5D-5L) enabling participants to rate their level of impairment across mobility, self-care, usual activities, pain/discomfort, and anxiety/depression and give a global health rating on a visual analogue scale (EQ-VAS), the EQ-5D-5L was found to be a highly reliable and valid measure of the quality of life in older people with mild to moderate dementia^27^; and (4) individualised mobility goal attainment will be measured with the Goal Attainment Scale (GAS) where participants and/or their ‘person responsible’ will identify a key goal related to physical functioning, the GAS is recommended as a measure of relevant person-centred outcomes in the evaluation of complex interventions in older people.^28^

*Cost-related outcomes* will include intervention delivery costs (from study records, including staff salary, travel and equipment costs) and health service utilisation costs (from calendars and aged care documentation records) during the trial period.

*Implementation outcomes and determinants* will use quantitative and qualitative methods to gather data on the implementation of the intervention and to inform future implementation and scale-up if found to be successful. Implementation outcome measures included are: (1) reach (proportion of aged care recipients who were successfully screened, consented to participate and received the intervention with an exploration of their representativeness), (2) fidelity (the extent to which the different components of the telehealth intervention are delivered per protocol and monitored through audits of study-specific checklists), (3) dose delivered (the number and duration of physiotherapy telehealth and coach sessions provided to participants plus total exercise dose over the trial period), and (4) adoption (proportion of facilities who participate and factors associated with uptake). Implementation determinants related to feasibility are: (1) proportion and representativeness of participants that completed the intervention at 6 months; (2) proportion and representativeness of available participants that completed the primary outcome at 6 months, and (3) adherence to the prescribed dose (percentage of participants recording 2 hours of exercise per week on their exercise diaries over the trial period).

The acceptability, barriers and facilitators for implementing this telehealth exercise and education programme for older people will be explored with participants, physiotherapists, aged care workers and aged care service managers via surveys and semistructured interviews.^29^ Up to 20 participants receiving the TOP UP intervention will be invited to participate in the interviews. We will sample participants purposively for maximum variation in (1) residence: home or residential aged care, metropolitan and regional, (2) age, (3) engagement with the intervention (as judged by the TOP UP coaches), and (4) sex: aiming for a distribution that reflects the male/female ratio in the intervention arm of the trial. We will interview up to 10 TOP UP coaches, 6–8 aged care managers and 6–8 physiotherapists involved in intervention delivery based in a range of aged care services and locations.

*Adverse events* such as a fall, musculoskeletal injury, or cardiovascular event that may, or may not, be related to the intervention but occurs while the person is participating in the intervention will be monitored via records kept by our aged care partners and by the participants in their self-report exercise diaries at 6 months. This information will be reported in any publications. A serious adverse event (SAE) will be defined as an incident that occurs while the person is participating in the intervention resulting in serious injury, hospitalisation or death.

If a SAE occurs, the research manager will notify the Data and Safety Monitoring Board (DSMB). The DSMB, consisting of two independent clinical experts and a statistician, will be convened to monitor SAE within 48 hours, where policies and procedures around the programme will be reviewed, and recommendations implemented. This assessment will guide the continuation of the research, including a temporary halt or early termination of the trial, ensuring ethical conduct of the study.

### Data management

We will use a custom-built and secure REDCap database. All study documentation will be stored securely in either locked filing cabinets (paper files) or electronically (electronic database files) with access granted only to authorised study team members. Logic and range checks will be used to minimise data entry errors and to identify missing data and other problems. To ensure confidentiality, the final dataset will contain deidentifiable information only. All publications associated with the study results will involve deidentified data to maintain participant confidentiality. Demographic information linking the participant to the data will be stored in a separate file. Only the principal investigator will have access to this information after the study.

### Sample size

A total of 240 participants (120/group) will provide 80% power to detect a 0.9 point between-group difference in 12-point SPPB scores at 6 months (assuming SD of 2.8, p=0.05, and 20% dropouts). A 0.5 point between-group difference in SPPB is considered to be clinically significant.^30^ This sample size is expected to be sufficient to detect between-group differences of 10–15% for the secondary outcome measures. These calculations used the *sampsi* command in Stata V.13 and PASS V.13 and allow for a 20% loss to follow-up.

### Data analysis

The primary analysis will be based on an intention-to-treat approach. The primary effectiveness outcome will be assessed as a change in participants' mobility using the SPPB. It will be treated as a continuous variable. The effect of group allocation on the outcome at 6-month follow-up will be analysed using linear regression models with baseline scores entered into the linear regression model as a covariate. Alternatively, if distributions are overly skewed (on visual inspection of histograms), the change scores (post-pre) will be analysed. The number of falls per person year will be analysed using negative binomial regression models to estimate the between-group difference in fall rates after 6 months with exposure entered as the offset variable.^31^ The other secondary continuous outcomes (five times sit-to-stand test and EQ-5D-5L) will be analysed using regression models with baseline variables as covariates as described for the primary outcome.^31^ Ordinal regression will be used to compare groups on the GAS.

Prespecified subgroup analyses will use interaction terms (group × outcome) in the model to explore whether there is a differential effect of the intervention in residential aged care facilities versus community-dwelling residents and in those with more marked cognitive impairment. Secondary analyses using causal modelling maybe conducted to establish intervention effects in people with greater exercise dose.^32^

Cost-effectiveness will be explored as the cost per extra person avoiding mobility deterioration as defined as an improvement or no change in the 12-point SPPB score between baseline and 6 months. Cost-effectiveness analysis may also be conducted on falls (ratios will be calculated relative to the control group for the incremental cost per fall avoided per person) and the quality-adjusted life year (QALY) gained (calculated from EQ-5D-5L). Bootstrapping will estimate a distribution around costs and health outcomes and calculate the CIs around the incremental cost-effectiveness ratios. The results will be plotted on the cost-effectiveness plane, and as cost-effectiveness acceptability curves. These methods have been used previously when calculating the incremental cost-effectiveness ratios for exercise programmes for older people.^33^

A detailed analysis of the implementation outcomes relating to the intervention’s reach, fidelity, dose delivered, adoption, feasibility and exercise adherence measures will be conducted. This data will be presented numerically and descriptively.^34^ Thematic analysis will be conducted for interview data.^35^ We will use the NASSS framework (Nonadoption, Abandonment and Challenges to the Scale-Up, Spread and Sustainability of Health and Care Technologies) to provide a conceptual ‘lens’ that will inform our implementation analysis. NASSS consolidates multiple implementation frameworks and empirical studies, targeting key issues relating to implementation and uptake of telehealth at the microlevel of individual staff and consumers, the mesolevel challenges of organisational engagement and adoption, and macrolevel policy and regulatory factors.^36^

### Patient and public involvement

The rationale, trial design and intervention content were informed by older people receiving aged care services and their advocates, aged care physiotherapists and aged care service providers via several online forums and many formal and informal meetings and conversations. Staff of aged care service provider organisations assisted with recruitment of participants for the research through direct communications within their organisations.

### Governance

The trial will be governed by a project steering committee, consisting of representatives from our aged care partners not directly linked to research activities, an independent consumer advocate and the study investigators. The committee will be chaired by a member of the research team. This committee will facilitate consumer and aged care input into the study, oversee the study protocol and data analysis, support results translation and be involved in the development of the resources at the end of the study period.

## Conclusion

This study addresses the international public health challenge of preventing mobility decline in older people. Deteriorating mobility is common and costly in older people receiving aged care services. The increasing cost of ageing and dementia care services means that there is an economic imperative for governments and service providers to support treatment innovations such as telehealth to ensure that older people have equitable access to appropriate and effective care.^37^ This study is the first known randomised controlled trial to rigorously examine the evidence about the effectiveness, cost-effectiveness and implementation of a telehealth physiotherapy-led exercise programme to improve mobility, reduce falls and improve the quality of life in older people receiving aged care services in their home or residential aged care.

## Data Availability

Data are available upon reasonable request.

## Data availability statement

Data may be made available on reasonable request.
